# Control Architecture for Connected Vehicle Platoons: From Sensor Data to Controller Design Using Vehicle-to-Everything Communication

**DOI:** 10.3390/s23177576

**Published:** 2023-08-31

**Authors:** Razvan-Gabriel Lazar, Ovidiu Pauca, Anca Maxim, Constantin-Florin Caruntu

**Affiliations:** Department of Automatic Control and Applied Informatics, “Gheorghe Asachi” Technical University of Iasi, 700050 Iasi, Romania; lazar.razvan@ac.tuiasi.ro (R.-G.L.); pauca.ovidiu@ac.tuiasi.ro (O.P.); anca.maxim@ac.tuiasi.ro (A.M.)

**Keywords:** control architecture, connected vehicle platoons, V2X communication, CACC, DMPC

## Abstract

A suitable control architecture for connected vehicle platoons may be seen as a promising solution for today’s traffic problems, by improving road safety and traffic flow, reducing emissions and fuel consumption, and increasing driver comfort. This paper provides a comprehensive overview concerning the defining levels of a general control architecture for connected vehicle platoons, intending to illustrate the options available in terms of sensor technologies, in-vehicle networks, vehicular communication, and control solutions. Moreover, starting from the proposed control architecture, a solution that implements a Cooperative Adaptive Cruise Control (CACC) functionality for a vehicle platoon is designed. Also, two control algorithms based on the distributed model-based predictive control (DMPC) strategy and the feedback gain matrix method for the control level of the CACC functionality are proposed. The designed architecture was tested in a simulation scenario, and the obtained results show the control performances achieved using the proposed solutions suitable for the longitudinal dynamics of vehicle platoons.

## 1. Introduction

Nowadays, with the ever-increasing number of vehicles on the highways, there is a stringent need to improve the driving experience quality through autonomous driving, by making use of the available vehicle connectivity. There is much interest in the research community to explore this topic, a testimony given by the following highly cited review works. In [1], a survey on the control of connected and automated vehicles (CAVs), with emphasis on control solutions for improving the energy efficiency of different powertrain architectures is given. In [2], a comprehensive survey on urban traffic signal control for CAVs, with both deterministic and stochastic approaches, is provided.

A typical control framework for connected vehicles (CVs) is given by the connected cruise control (CCC) architecture, which is suitable for a vehicle group consisting of both autonomous and human-driven vehicles, connected with a vehicle-to-vehicle (V2V) communication network [3]. In [4], a fuzzy support vector machine (SVM) method for CCC, using radar and V2V communication to detect the lane change of a side vehicle, is proposed. In [5], a deep reinforcing learning (DRL) solution to solve a CCC problem with communication delays and dynamic traffic changes is given. In [6], a nonlinear range policy for a CCC application with merging capabilities is provided.

Vehicle platoons are control applications for groups of CVs, which are designed using the advantages of vehicle-to-infrastructure (V2I) and V2V connectivity. To perform a desired common task for the entire platoon, each vehicle needs to exchange the relevant local measured data with the other participants. Usually, a vehicle platoon task is to travel with a velocity imposed by the leader vehicle of the platoon, while maintaining a desired safe distance between the follower vehicles [7]. One of the most promising functionalities for CAVs is cooperative adaptive cruise control (CACC). The most defining things for CACC are the use of sensors and communication technologies. As an extension of adaptive cruise control (ACC), in CACC systems, CAVs use V2V communications to exchange information with other CAVs in an autonomous manner, as well as V2I to provide information on traffic conditions and traffic management [8].

There are several design frameworks that have recently been researched for the platoon formation of multiple CVs, such as non-cooperative differential games [9], estimation of the communication delays via an adaptive switched predictor [10], model-based predictive control (MPC) with switching communication topology [11], robust feedback control [12], or a cooperative adaptive sliding mode [13], among others. In [14], an LMI-based optimisation problem for a cooperative optimal control method for a CAV platoon is given. In [15], a consensus-based impulse control method, which simplifies the communication exchange in a platoon of CVs, is proposed. In [16], security for the distributed platooning control of CAVs, subject to denial-of-service (DoS) attacks, is discussed. In [17], an ACC strategy for CAVs in a platoon formation subject to cyber-attacks and communication delays is given.

However, in the state-of-the-art literature, there are few studies that detail the design of a complete control architecture for a connected vehicle group. Thus, this work presents a detailed survey regarding the components required by a control architecture for a vehicle group, by describing the multitude of options available in terms of sensors, control, and communication requirements. Moreover, the proposed study of the control architecture can be used as a tutorial in designing a control solution for an automated vehicle group.

The main contributions of this paper are the following:

- A detailed survey was carried out on the solutions available in the literature of general control architectures for the CAV platoon, from the point of view of the constructive levels, the elements that define each level, and the links between them;
  Moreover, in relation to other works on this topic [1,18,19], this paper presents a more detailed description of the sensors and V2V communication standards essential for the CAV concept;
- Starting from the proposed architecture, a suitable control architecture is defined for a platoon of connected vehicles based on the CACC strategy, by presenting the necessary sensors, the suitable types of communication means, and the control solutions;
- Finally, two control methods are proposed for the longitudinal dynamics suitable for a CAV platoon framework. Thus, a state-space distributed model-based predictive control (DMPC) method suitable for vehicle platooning is described and tested. Moreover, a second control method is proposed, designed using off-line optimisation to compute a feedback gain control matrix. Both methods are compared using a CAV platoon application.

The remainder of this paper is structured as follows. Section 2 deals with a general presentation of architecture design for connected vehicle platoons, by describing the main subsystems, their purpose, and component elements. In Section 3, different aspects of the vehicle communication systems are presented, synthesised under intra-vehicle communication and inter-vehicular communications. Section 4 presents the proposed control architecture for a specific case involving a CACC strategy and model used to describe the longitudinal dynamics of a vehicle platoon. Section 5 illustrates the simulation results obtained using the proposed control solutions, and Section 6 includes a thorough analysis based on their resulting performances. In the last section, one can find the conclusions and future research directions.

## 2. Architecture Design for Connected Vehicle Platoons

The control architecture design defines the necessary stages in the transformation of an ordinary car into a connected one by adding additional components, including different sensors that allow the vehicle to detect the environment and communicate with other traffic participants and with the intelligent infrastructure, as well as adequate control strategies for controlling the vehicle’s mobility.

**Figure 1:**
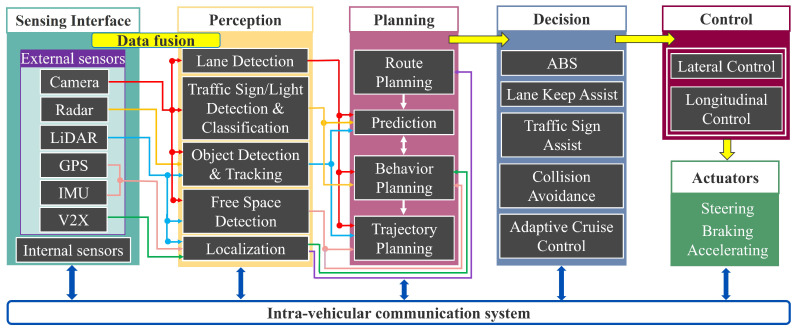
Example of a general control architecture.

A general control architecture is presented in Figure 1, where the communication process between the most important subsystems of the architecture is illustrated. At a high level, the architecture has five subsystems, responsible for defining the autonomy process of a connected vehicle, such as sensing interface, perception, planning, decision, and control. The proposed architecture is used by each vehicle regardless of the position within the platoon. Depending on the type of the vehicle (leader or follower), the general control architecture presented can be used differently, from the point of view of the component elements and the functions performed by each of its subsystems.

According to the work in [20], these subsystems, which are described in detail in the following subsections, have the purpose of (1) retrieving information from the real environment around the vehicle (i.e., the sensing interface), (2) fusing the data with the purpose of detection and localisation (i.e., perception), (3) choosing the route (i.e., planning), (4) predicting the behaviour of other traffic participants and planning the optimal trajectory (i.e., decision), and based on the taken decision, (5) controlling the vehicle by operating the various responsible actuators (i.e., control). In order to exchange the necessary information between them, these subsystems are connected with a car-level communication system, more precisely, the intra-vehicular communication network. For the exchange of information between several connected vehicles, each with its own control architecture, an inter-vehicular communication network is used, synthesised under vehicle-to-everything (V2X) [21].

### 2.1. Sensing Interface

**Figure 2:**
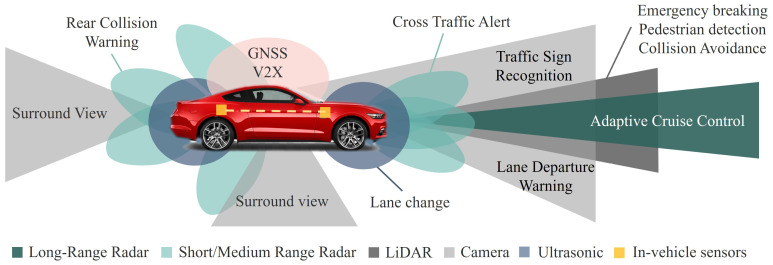
Typical types of sensors and their functionalities.

This subsystem shows how the information is captured from the vehicle’s environment, such as the detection of its position in relation to the surroundings, but also information about the other traffic participants. The sensing interface consists of different sensors for data collection. These sensors can be classified into two categories: (1) internal sensors that provide information only about the state of the vehicle and (2) external sensors used to capture data from the outside of the vehicle. All of them are better exemplified in Figure 2, where one can see the sensors’ position, the coverage area, and the performed functions.

#### 2.1.1. Literature Review

In the following, the most important external sensors are presented in detail:

(1) The camera is one of the key sensors of a vehicle, used for perceiving the visual environment, lane and traffic sign recognition, object tracking, and much more [22]. Cameras may be mono, stereo, or full surround, and placed in areas such as dashboards and windshields. These types of cameras used for autonomous vehicles are described in detail in [7]. Depending on the quality of the lens, the maximum working distance of the camera is around 250 m. The main advantages of a camera are related to the accuracy of the colour distribution, the contour of the surroundings, and the texture [23]. As a disadvantage, cameras are sensitive to low-intensity lights and can be affected by weather conditions [24].
(2) Radar is the most common sensor used in vehicles to identify and locate objects in the presence of various interferences, such as noise, clutter, and jamming [25]. To measure the distance, the time Of flight (TOF) method [26] is used, whereas to measure the relative velocity, the Doppler shift [23] is used. Thus, radars lend themselves very well for obstacle detection [27] and pedestrian and vehicle recognition [28,29]. Also, some functionalities of the radars are blind spot detection, rear collision warning, emergency braking, and cross-traffic alert. Radar sensors operate in the millimetre-wave (mm-Wave) spectrum, using different frequency bands, such as 24, 60, 77, and 79 GHz, being able to measure a range from 5 to 200 m [30]. Depending on the type of radar and the application for which it is used, radar sensors are divided into short, medium, or long-range ones. The most important characteristics of the types of radars used in the automotive field are presented in [25]. Radar sensors also offer the benefits of high availability and low cost [31]. Moreover, compared to cameras, they are less affected by the weather and the low lighting environment [26]. Disadvantages include lack of precision, receded field of view (FOV), and the production of false positives by rejecting emitted signals [32].
(3) A light detection and ranging (LiDAR) sensor is a technology used to determine precise information about the distance and size of objects [33]. It uses a remote sensing technique, producing pulses of infrared or laser light and measuring the time it takes for the pulses to be reflected [34], a principle known as TOF, and it is similar to how the radar sensor operates. The range of LiDAR is about 200 m on average [35], using 905 nm and 1550 nm spectra [36]. There are different types of LiDAR sensors, these being 2D, 3D, and solid-state [37]. The general specifications for each type of LiDAR sensor are presented in [34]. Compared to the camera, the LiDAR sensor has better detection capabilities in terms of range, with bad weather and low lighting affecting this sensor less than the camera [26]. Compared to radar, it has a higher accuracy and precision, but also a superior 3D perception competence. As a disadvantage, the LiDAR sensor is affected by severe weather conditions, such as snow, fog, and rain [38]. Moreover, in terms of cost and low availability, the LiDAR is less competitive than the other two types of sensors.
(4) An ultrasonic sensor is the most diligent and cheap sensor, used for short-range obstacle detection, proximity sensing for lane change, and parking functions [39]. These sensors use ultrasonic waves to measure the distance to objects by calculating the TOF of the emitted wave. These sensors operate in the 20–40 KHz range, with a detection range of generally less than 11 m [40], and are used at low speeds. Also, these sensors are easier to implement, and work satisfactorily in bad weather conditions and in dusty environments [41]. The main disadvantages are the disturbances in the sound waves and the tendency to produce false positives in the measurements, as well as the need to use multiple sensors to obtain a complete view; thus, mutual interference is produced between them [42].
(5) Global Positioning System (GPS) and inertial measurement unit (IMU) technologies are used for navigation and localisation purposes, by determining the exact position of the vehicle and helping it to navigate. The GPS is a system used to obtain information about geolocation, speed, and time, each vehicle containing a GPS receiver that connects to GPS satellites [43]. The position of the vehicle is given by the GPS coordinates, but the accuracy with which these are extracted depends on several factors. Therefore, position errors can be obtained with an average value of 3 m, and with a deviation of 1 m [44], and can reach up to 20 m depending on the environment. In urban environments, the GPS performances are lower [45]. It presents advantages in terms of cost and the way of managing the accumulation of errors over time. The disadvantages would be related to precision, which is reduced to one metre for current vehicles, but also the inability to operate in environments where the view of the sky is obstructed, such as tunnels [26]. An IMU is an electronic device that measures and reports the body’s specific force, angular rate, and sometimes the magnetic field surrounding the body, using a combination of accelerometers and gyroscopes, sometimes also magnetometers [46]. Therefore, with the help of these data, the linear velocity and angular positions for the vehicle can be calculated. The IMU sensor can be combined with the GPS, as a complementary sensor, because the IMU can not give the position error by itself, but also for the performance qualities of the IMU sensor in tunnels [47]. Moreover, to improve the estimation of the vehicle’s position, different techniques are used in which GPS and IMU data are fused [48].

#### 2.1.2. Summary

Table 1 illustrates the comparison between external sensors from the point of view of the most relevant metrics. Thus, it can be concluded which of them lends itself best depending on the functionality chosen for CAVs from the platoon.

Due to the fact that the leader vehicle is in front of the platoon, it imposes the travel velocity and direction for all members, and has also the role of detecting the lanes and the various obstacles on the road. To fulfil the leader’s tasks, it can be equipped with the following sensors: (i) camera (for lane, traffic signs/lights, and obstacle size detections), and (ii) radar and LiDAR (for obstacle detection at large distances). In other words, because the follower vehicles require high accuracy for distance measurement, each follower can be equipped with radar, and with a camera for lane detection. The GPS and IMU sensors should be used by each vehicle from the platoon to measure their velocity and obtain their position.

### 2.2. Perception

The perception subsystem is composed of software elements that receive the information from the sensors and combine and structure it in a simpler form, in order to classify it. Sensor fusion is a necessary process for this stage, and involves the combination of information from all the available sensors in the vehicle [49]. Thus, a complete assessment of the environment can be carried out and more precise information can be obtained.

#### Literature Review

In practice, different algorithms are used for the fusion process, such as Kalman and Bayesian filters [20]. In [23], several data fusion methods are presented, based on the following strategies: discernible units, complementary features, target attributes, and the decision making of different sensors.

The perception subsystem is in charge of two key tasks: the localisation of the ego vehicle and the detection of other traffic participants and other elements of interest from the surrounding environment. Within the localisation task, the location of the vehicle relative to a map is computed. More precisely, the vehicle’s position is determined using data received from different sensors, such as GPS, IMU, LiDAR, and V2X communication, combined with the use of maps. The work in [50] presents a localisation system for urban and indoor scenarios, where LiDAR, IMU, and GPS sensors are integrated. A multitude of combinations of different sensors for data fusion in localisation and mapping are presented in [51], with an emphasis on limitations without fusion and fusion advantages. The detection process uses camera, radar, LiDAR, and ultrasonic sensors to collect the necessary data for the identification and classification of various elements of the vehicle’s external environment. In [52,53], a fusion between camera and LiDAR used for pedestrian detection is given, whereas in [54,55], the same is employed for road detection. Moreover, for vehicle and lane detection, information from camera and radar are used in [56]. The perception subsystem is used by both leaders and followers to fuse sensor measurements.

### 2.3. Planning and Decision

The planning subsystem uses information from the perception subsystem to find the most suitable route for the vehicle, from the origin to the destination, both for short-term and long-term planning. The GPS navigation system has the role of a global planner, being used to plan routes, but considering the current requirements, it does not ensure the safety of the user [57]. In this context, according to Figure 1, a traditional planner structure for a self-driving car consists of a route planner, behaviour planner, and trajectory planner.

The decision subsystem, which is the next step after planning, makes a decision and sends all the information to the control subsystem by assuming a compact data form received from the previous subsystems. A number of the decisions available for connected vehicles are illustrated in Figure 1, such as anti-lock braking systems (ABS), lane keep assist (LKA), traffic sign assist (TSA), collision avoidance (CA), adaptive cruise control (ACC), and more. The decision-making process is based on the information from the previous subsystems available at the current moment, but also uses past information. Furthermore, real-time data from maps, traffic models, and additional information from the driver are also used as information. Thus, depending on the type of decision chosen, based on the collected information, it is forwarded to the control subsystem, which in turn will choose the appropriate control type and optimal method.

#### Literature Review

Route planning, at a general level, involves the use of a route planner, whose purpose is to identify the path that a vehicle must travel between two cardinal points. Moreover, route planning includes several dynamic parameters, such as congestion level, spontaneous indicators, meteorological conditions, and others [58]. An optimal route consists of a continuous adjustment of the planning process, in which the vehicles decide their routes in an adaptive manner. Considering the unexpected changes that may appear along the route (e.g., traffic barricades or lane obstructions), an efficient planning subsystem employs dynamic optimisation techniques at each discrete moment of time. For this type of planning, updated maps and data provided by the localisation stage from the previous subsystem are basically used. In the case of connected vehicles, a distributed route design strategy is used, where each vehicle collects its own traffic data and calculates its own route, thus improving the overall calculation time for the route planner [59].

Behaviour planning involves the use of a behaviour planner, which is closely related to a predictor. The prediction component evaluates the behaviour of other traffic participants, such as vehicles and pedestrians, but also other intervening obstacles, in order to obtain risk and road traffic management [60]. Moreover, another source of information for this planner is sensor fusion data from the perception subsystem. As such, the behaviour planner uses the information from lane detectors, traffic lights, and traffic signs, as well as detected objects, but also information from the localisation part. All this information is used to plan their own safe handling behaviour. Therefore, having all these data as inputs, certain decisions are issued for the vehicle, such as maintaining or changing lanes, maintaining the current distance from the vehicle in front, and maintaining the speed by braking or accelerating.

Trajectory planning uses a trajectory planner in order to generate a series of trajectories based on the behaviour planner, taking into account several aspects, such as driver comfort, various road limitations, and vehicle dynamics [61]. The most used methods to design the trajectory planner are based on polynomial equations [62,63], Bézier curves [64,65], and MPC algorithms [66,67]. Thus, taking into account the previously mentioned aspects, the desired trajectory is determined and sent to the decision subsystem [68].

The planning subsystem is mainly intended for the leader vehicle; it computes the global route using localisation functionality based on GPS and V2X communication. Based on the information from the perception subsystem, the leader determines the general behaviour of the platoon by choosing the most suitable action. After that, it must inform the followers about the chosen decision (maintaining or changing lanes, maintaining the speed by braking or accelerating, and more). Finally, based on the previously mentioned information, the leader uses trajectory planning to compute a path so that the platoon can follow the global route and avoid collisions that may occur.

The follower vehicles receive the decision taken by the leader, and depending on this, the following cases result: (i) maintaining or reducing the speed to ensure an imposed distance to the vehicle in front (longitudinal dynamics); and (ii) maintaining or changing lanes to minimise the lateral position error between followers and the vehicle in front.

### 2.4. Control

The control subsystem receives its task and all related information from the decision subsystem. Thus, starting from the desired trajectory determined by the planning subsystem, and the imposed driving action, implements the best decision for the vehicle. Following the description from Figure 1, once the decision is taken, an appropriate control strategy is selected.

#### Literature Review

Let us assume that a collision avoidance action is requested. This can happen if an unexpected obstacle is detected on the travel lane in front of the vehicle. Prior to this decision, at the stage of the planning subsection, the trajectory planner computes the optimal trajectory to avoid the obstacle, starting from the current position measured by the sensors to the final position for the vehicle, which usually is on the neighbouring available lane. In the control subsystem, using this information as input data, the lateral control strategy is implemented. In this case, the end result of the control action is a steering movement, i.e., the vehicle’s wheels are moved with the desired steering angle while following the planned trajectory [68]. The lateral control supposes that vehicles are equipped with GPS and IMU sensors to measure their position and orientation; also, the vehicles have to be equipped with LiDAR and camera sensors to measure their position and orientation with respect to the neighbour vehicles or an obstacle. In the case of an ego vehicle, the lateral control function uses the measurements of these sensors and inputs received from the planning and decision subsystems to steer the vehicle so that it follows the imposed trajectory and avoids collision with obstacles or other vehicles. In the case of a vehicle group (e.g., platoon), there are three preferred approaches: (i) follower vehicles do not use information about the vehicle in front and they only follow the road; (ii) a follower vehicle receives from the vehicles in front information about their lateral references and uses it to determine its own future trajectory; and (iii) the case in which the follower vehicle is following the lateral trajectory of the vehicle in front. In the last two cases, the use of vehicle communication can improve performances due to the fact that the follower is not using only measurements from its own sensors, but also information received from its neighbour vehicles. The most used approaches for lateral control are based on the MPC strategy [69,70,71], LQR algorithm [72,73], adaptive control [74], and optimal control [75]. The lateral control of the leader involves steering the vehicle according to the trajectory from the planning subsystem. Also, the follower vehicles use lateral control to track the trajectory of the vehicle in front and to maintain the lanes (if no other functionality is chosen, e.g., collision avoidance).

Moreover, when ACC-based travel is decided, the most suitable control strategy is longitudinal control. This means that the vehicle must travel with an imposed longitudinal velocity (i.e., cruise control) while maintaining a safe distance with respect to the vehicle in front (i.e., headway control). Here, the control action is either braking, if the current velocity is greater than the desired velocity, or accelerating, if the measured velocity is lower than the imposed target [76]. The cruise control (CC) functionality is specific for an ego vehicle or the leader vehicle from a platoon. The vehicles use sensors like GPS and IMU to measure their velocity, which is used afterwards to compute the error to the imposed velocity. The longitudinal controller uses these measurements and errors to calculate the control inputs that command the brake or acceleration. In the cases of ACC functionality, follower vehicles have to use sensors like radar or LiDAR to determine the distance between vehicles. Moreover, if the vehicles can exchange information through communication networks about their velocity, acceleration, or position, then the CACC functionality can be used to ensure improved performances obtained by the ACC. The most used approaches for the longitudinal control are also based on the MPC algorithm [62,77,78], LQR [79], and PID controllers [80]. The advantage of using the MPC strategy and vehicle communication compared to the other methods consists of the possibility to use the future actions’ predictions of a neighbouring vehicle. The leader vehicle uses longitudinal control to travel with the imposed velocity, and the follower vehicles use it to maintain the imposed distance from the vehicle in front.

## 3. Vehicle Communication

This section presents the different aspects of the vehicle communication systems by introducing several communication standards used for the exchange of information between the vehicle components (i.e., intra-vehicle communication), as well as for the inter-vehicular communications, between the vehicle and other traffic participants or intelligent infrastructure, known as V2X communication.

### 3.1. Intra-Vehicle Communication

Intra-vehicle communication is an absolutely necessary requirement to take into account in the development of new model cars. Thus, for proper vehicle operation, strict information must be exchanged in real time between different nodes/modules. Depending on the communication architecture, the amount of data to be transmitted, bandwidth, reliability, and security, several networks can be distinguished.

#### 3.1.1. Literature Review

The most important intra-vehicle protocols are presented in the following:

(1) The controller area network (CAN) protocol is an automotive-specific bus standard, usually used for powertrain and body control applications. Thus, from the data rate point of view, two networks can be distinguished: (i) high-speed CAN (500 Kb/s) for real-time control for chassis and power-train electronic control units (ECUs), and (ii) low-speed CAN (125 Kb/s) for body and comfort electronics. The CAN is a multi-master serial bus that uses multiplexed communication between several ECUs in the vehicle [81]. Related to CAN arbitration, bus access conflicts are resolved by bit-level arbitration using the carrier sense multiple access with bitwise arbitration (CSMA/BA) technique. The CAN also contains five mechanisms for error detection, three for the message level: cyclic redundancy check (CRC), frame check, and acknowledgement (ACK) bit; and two for the bit level: bus monitoring and bit stuffing [82]. The advantages of CAN are reliability, robustness, low cost, high flexibility, and low network complexity. As disadvantages, it would be that it is not deterministic, it is not suitable for safety-critical applications, and it has a low bandwidth [83].
(2) The local interconnect network (LIN) is a low-cost network used for simple, less time-critical applications, especially used for connecting sensors and actuators. The LIN protocol uses the master–slave architecture; the master sends a frame header and the slave node must respond with a frame response. For low-cost requirements, a single wire is used at the physical level, thus resulting in a limited data rate of 19.2 Kb/s [84]. To detect incorrect messages in the network, LIN uses parity bits and checksum. The advantages of the LIN network are related to the ease of use, low implementation costs, and its deterministic characteristic when compared to other networks. As disadvantages, it is not as reliable as CAN, has a lower bandwidth, and less effective bus access, and cannot be used for time-critical applications [85].
(3) The FlexRay protocol was developed by the FlexRay consortium, and it is used for time-critical applications in the advanced chassis control area [86]. During data transmission, each node uses two parallel communication channels, the exchange of information being performed based on a communication schedule. Regarding the bus access principle, two methods are used: time division multiple access (TDMA) and flexible TDMA (FTDMA) [87]. A FlexRay frame consists of three parts: the header, the payload segment, and the trailer CRC. For error protection, checksums and redundancy mechanisms are used [88]. The advantages of the FlexRay protocol are its flexibility, higher data rate, and deterministic behaviour. Moreover, it offers constant latency and scalable fault tolerance, which makes it suitable for “drive-by-wire” applications. The disadvantages of this protocol can be summed up in the very high implementation costs and the high complexity compared to CAN [89].
(4) The Media Oriented Systems Transport (MOST) protocol is a multimedia network developed for infotainment applications. The bandwidth is up to 150 Mb/s, supporting both synchronous and asynchronous transmission. The MOST network can manage up to 64 devices using a ring topology, which can be easily connected and removed using the plug-and-play functionality. Moreover, different end-user applications can be connected to this network, such as radios, GPS, and entertainment systems [90]. Although this protocol satisfies the requirements for infotainment applications, there is a limitation in terms of bandwidth for certain requirements [91].
(5) Automotive Ethernet is a communications bus that successfully serves high-bandwidth applications in the field of autonomous driving and connected cars. Ethernet technology has several uses besides in-vehicle communication, such as measurement and calibration, but also diagnostics over IP (DoIP) [92]. The Ethernet standards used for automotive requirements are 100Base-T1 and 1000Base-T1. Automotive Ethernet is implemented with a single twisted cable pair, obtaining data rates from 10 Mb/s to 10 Gb/s. Ethernet lends itself very well to the requirements of applications in the advanced driver-assistance system (ADAS) field, which requires the use of large bandwidth for the sensors used. Moreover, related to diagnostics, Ethernet has started to replace CAN, offering a much shorter time for flashing procedures [89]. Supporting a switched network technology, another advantage is the reduced cost of cabling. The main disadvantages are related to the high costs, resulting in a more expensive physical-level interface [90]. Besides this, Ethernet does not offer deterministic and real-time communication, and this is the main reason why automotive Ethernet does not completely replace the CAN protocol.

#### 3.1.2. Summary

A comparison of in-vehicle network protocols from the point of view of technical characteristics is illustrated in Table 2, where can be observed the essential aspects that can classify each protocol according to its advantages, as it can be concluded which is more suitable depending on the chosen architecture.

### 3.2. V2X Communication

V2X communication technology has major importance in the implementation of an intelligent transport system (ITS), offering a level of automated driving and intelligent mobility. Furthermore, this technology involves the exchange of information between a vehicle and other entities of the traffic system.

#### 3.2.1. Literature Review

**Figure 3:**
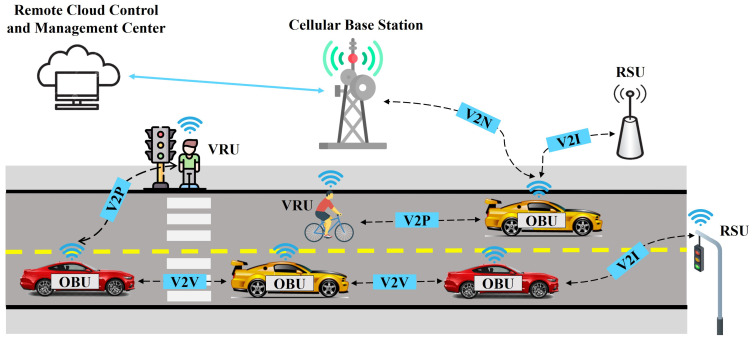
V2X communication modes.

The V2X includes four modes of communication: vehicle-to-vehicle (V2V), vehicle-to-infrastructure (V2I), vehicle-to-pedestrian (V2P), and vehicle-to-network (V2N) [93]. Each of these communication modes is illustrated in Figure 3 and exemplified in the following:

(1) V2V communication allows for the exchange of information between vehicles in proximity, exchanging useful information about vehicle location, traffic accidents, speed, and traffic dynamics [94]. Each vehicle is equipped with an on-board unit (OBU). Communication between vehicles is achieved by forming a mesh network and connecting them as nodes to the network [95]. Therefore, for the exchange of information between nodes, messages are used with the aim of creating a more efficient decision-making system. Thus, if used properly, V2V communication has the benefits of increased driver safety and road capacity, improving fuel efficiency, and preventing possible accidents [96].
(2) V2I communication refers to the exchange of information between the vehicle and various equipment installed on the road infrastructure [97]. V2I communication can be ad hoc, wireless, or bidirectional [98]. The vehicles collect information from a road side unit (RSU), which is a stationary unit installed along the roads. This information is used for traffic management [99]. Thus, useful information can be obtained about traffic congestion, available parking, the most efficient routes, and road conditions [100]. All these are used to obtain reduced fuel consumption, increase mobility, and reduce polluting emissions [22].
(3) V2P involves real-time, wireless communication between vehicles and vulnerable road users (VRUs), such as pedestrians, bicyclists, and more [101]. Each VRU has user equipment (UE), usually a mobile phone, which makes it possible to exchange information with vehicles. Thus, messages and alerts are sent about the location, speed, and direction of VRUs [102]. Using V2P, communication between vehicles and VRUs can be achieved even in unfavourable weather conditions [93]. Therefore, the benefits of this type of communication refer to the improvement of pedestrian safety and the reduction in traffic accidents in which VRUs are involved.
(4) V2N communication allows the vehicle to access the network, through a server, for various cloud-based services. This type of communication can be made directly between the vehicle and the network or indirectly through a node installed in the road infrastructure, depending on the distance between the vehicle and the network infrastructure [103]. Vehicles can receive broadcast alerts regarding various aspects of traffic, such as accidents ahead, traffic congestion, or support for planning the best route. All this leads to increased vehicle safety, better route planning, and better traffic efficiency [104].

V2V communication is used for all platoon members to exchange information related to position, velocity, acceleration, and decision. Moreover, V2P communication is also necessary for leaders and followers according to safety considerations for vulnerable road users. The V2I and V2N communications are used only by the leader vehicle to obtain information about traffic management, traffic congestion, road conditions, accidents ahead, and support for planning the best route.

From the point of view of the communication standards used for V2X communication, two important categories of communication technologies are distinguished: dedicated short-range communication (DSRC) and Cellular-V2X (C-V2X) technology. Each of these is presented in detail in the following:

(1) DSRC is a specific communication standard for V2X technology, which allows for wireless communication between connected vehicles, but also with road infrastructure. The DSRC involves short-range bidirectional wireless communication, and it is used for V2V and V2I communications [8]. The DSRC system is based on a series of IEEE and SAE standards. For the physical (PHY) and medium access control (MAC) layers, DSRC uses the IEEE 802.11p standard, for requirements related to authentication, data transmission, and high mobility challenges. The network and security services are defined in the IEEE 1609.x family of standards [105,106]. In the U.S., the Federal Communications Commission (FCC) has allocated for DSRC a 75 MHz spectrum, divided into 10 MHz channels, in the 5.9 GHz frequency band [8]. DSRC-based V2X is successfully used in applications such as traffic safety, traffic management, and commercial vehicle applications [107]. Thus, this standard comes with the following benefits: low latency, high reliability, data rates from 3 Mbps up to 27 Mbps, and ad hoc communications. Cooperative awareness messages (CAMs) and event-triggered warnings, i.e., decentralised environmental notification message (DENM)-type messages, were established in the IEEE 802.11p standard by The European Telecommunications Standards Institute (ETSI). On the other side, in the U.S., the basic safety message (BSM) set messages have been defined by the Society of Automotive Engineers (SAE) [108]. The CAM and BSM are periodical messages sent between vehicles and between vehicles and the infrastructure. These contain information about the status information on heading, speed, position, and acceleration. Moreover, for V2X applications, the transmission frequency of CAM messages is standardised between 1 to 10 Hz, and the broadcast rate of BSM messages is 10 Hz. The DENM messages are warnings transmitted in emergency situations. These are decentralised and information is transmitted directly between vehicles, without the involvement of a centralised infrastructure [109].
(2) C-V2X technology is based on cellular systems, merging the traditional V2X network with the cellular network [110]. According to the 3rd Generation Partnership Project (3GPP) unified global standards, this communication technology uses long-term evolution (LTE)–V2X for assisted driving and 5G New Radio (NR)–V2X for autonomous driving [111]. The working frequency for C-V2X is the same as in the case of the DSRC, operating in the 5.9 Hz frequency band, a band allocated for communications in the intelligent transportation system (ITS) area. LTE-V2X uses single-carrier frequency division multiple access (SC-FDMA) and supports 10 MHz and 20 MHz channels. The communication channel for LTE-V2X uses resource blocks (RBs) of 180 kHz; this implies 12 subcarriers of 15 kHz each. Moreover, from the point of view of time, the channel is divided into sub-frames of 1 ms [112]. LTE-V2X uses communication modes 3 and 4 for resource allocation. The initial advantages of LTE-V2X were improving road safety and reducing traffic congestion. This is possible by periodically broadcasting a CAM message between connected vehicles and LTE-V2X supporting in-coverage, out-of-coverage, and partial-coverage scenarios.
  The 5G NR-V2X technology started to be developed from Release 16, coming as a complement to LTE-V2X. For 5G NR, two frequency ranges are defined in which it can operate: frequency range 1 (410 MHz–7.126 GHz) and frequency range 2 (24.25–52.6 GHz). This results in bandwidth for the channel in both bands of 10, 20, 30, and 40 MHz. Besides these, 5G NR-V2X supports various frequency division multiplexing (OFDM) methodologies [113]. For 5G NR-V2X, two communication modes are defined, as in the case of LTE-V2X, mode 1 and mode 2. The advantages of 5G NR-V2X technology include increased capacity and speed, as well as reliability, but also a considerable decrease in latency [114]. The C-V2X uses two complementary transmission modes: the Uu and PC5 interfaces. Modes 1 and 3 correspond to the Uu interface, this being a traditional radio interface that accesses terminals through a base station, using uplink (UL) and downlink (DL) transmissions. Within Uu, C-V2X applications operate in traditional mobile broadband licensed spectrum. This interface is used in V2N communication, for long-range applications. The PC5 interface corresponds to modes 2 and 4 and involves direct communication between traffic entities, unassisted by the base station. It is used for V2V, V2I, and V2P communications, the exchange of information being carried out with the help of sidelink (SL) transmission. Within PC5, C-V2X applications operate in the 5.9 GHz ITS band for short-range applications, on a distance of less than 1 km [115].

#### 3.2.2. Summary

The technical characteristics of DSRC and C-V2X standards are illustrated comparatively in Table 3, where can be observed the properties of each communication technology: IEEE 802.11p, LTE, and 5G NR. Therefore, depending on the V2X communication requirements for each use case, the appropriate communication standard can be chosen.

## 4. Cooperative Adaptive Cruise Control for Vehicle Platoon

Interconnected vehicle systems are built around the ability of multiple vehicles to establish a local network and communicate with one another their mobility characteristics so that cooperative maneuvers can be performed such as maintaining lanes, maintaining a constant speed, changing lanes, and many others. In recent years, cooperative adaptive cruise control (CACC) has emerged as a promising technology in vehicular safety applications. CACC-based platoons involve a group of vehicles that are connected through wireless communication and are capable of performing coordinated driving manoeuvres, such as acceleration, deceleration, and lane changing. The use of CACC in platooning applications has been shown to improve traffic efficiency, reduce fuel consumption, and enhance safety by mitigating the effects of human error [8].

**Figure 4:**
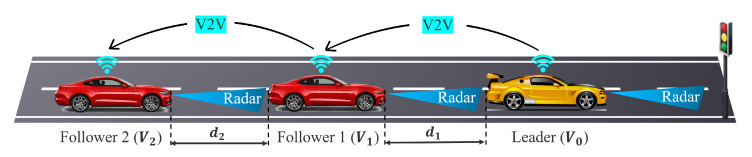
Cooperative adaptive cruise control for a vehicle platoon.

This section presents a proposal for a control architecture targeting a specific case involving a CACC strategy for a vehicle platoon, as illustrated in Figure 4. The topology used is predecessor–follower; thus, the vehicles periodically transmit their current state information, such as location, speed, and acceleration. The proposed control architecture that implements the CACC functionality has the following components:

- Sensors:
  - The leader is equipped with a long-range radar for obstacle detection;
  - The follower vehicles are equipped with a short-range radar to measure the distance to the preceding vehicle;
  - Each vehicle is equipped with a camera for lane and obstacle detection, and GPS and IMU to determine the position and velocity of the vehicle.
- Vehicle communication:
  - The communication channel consists of a V2V link between the vehicles. Thus, vehicles are equipped with DSRC technology for short-range communication using CAM messages. These are sent with a frequency of 10 Hz, with each vehicle sending 10 messages per second, which is the minimum required by the CACC functionality [116]. The bitrate for CAM messages is set to 6 Mbit/s, which means an optimal value for vehicular scenarios [117];
  - For each vehicle, the data from the sensors are processed and then transferred to the vehicle’s central control unit for fusion using a CAN bus. Also, the automotive Ethernet bus is preferred for the camera sensor according to its required high bandwidth.
- Control solutions:
  - Specifically for lateral control, vehicles can use an LQR algorithm to implement the lane keep assist functionality;
  - For the longitudinal control, two methods based on the DMPC algorithm and feedback gain matrix are proposed.

In what follows, the modelling method and the two control strategies proposed for the longitudinal dynamics are detailed.

### 4.1. Vehicle Platoon Modelling

The leader vehicle (denoted with $V_{0}$) is in front of the platoon and leads the platoon with a desired travel velocity. The follower vehicles are tracking the vehicle in front while keeping an imposed distance from it. Each vehicle uses GPS, IMU, camera, and LiDAR to measure the velocity, acceleration, position, and velocity errors, and receives information about the states of its in-front neighbour via V2V communication. Moreover, the control solution for CACC functionality assumes that in front of the leader is a “virtual leader” vehicle that is moving with the desired acceleration. In this way, the leader vehicle can be modelled as a follower that has to follow the virtual leader. The model that describes the longitudinal dynamics [118] is given by (1). This model describes the relationship between states of the vehicle, position and velocity errors and inputs. Moreover, the model illustrates the coupling between two consecutive vehicles from the platoon:

(1) $$\begin{array}{c} \left[\begin{array}{c} {\dot{e}}_{pi}\left(t\right)\\ {\dot{e}}_{vi}\left(t\right)\\ {\dot{a}}_{i}\left(t\right) \end{array}\right]=\left[\begin{array}{ccc} 0 & 1 & -\delta \\ 0 & 0 & -1\\ 0 & 0 & -1/\tau \end{array}\right]\left[\begin{array}{c} e_{pi}\left(t\right)\\ e_{vi}\left(t\right)\\ a_{i}\left(t\right) \end{array}\right]+\left[\begin{array}{c} 0\\ 0\\ 1/\tau \end{array}\right]u_{i}\left(t\right)+\left[\begin{array}{c} 0\\ 1\\ 0 \end{array}\right]a_{i-1}\left(t\right), \end{array}$$

where $e_{pi}$ represents the longitudinal position error of vehicle *i* to the vehicle in front i−1; $e_{vi}$ represents the velocity error; $a_{i}$ represents the acceleration; $u_{i}$ represents the input, i.e., acceleration request; δ=0.7 s represents time headway; and τ=0.1 s represents the time constant. Notice that for the leader vehicle i=0, the acceleration $a_{-1}$ represents the virtual leader’s imposed acceleration $a_{r}$.

To control the vehicle platoon, this study proposes two control solutions based on the distributed model-based predictive control (DMPC) strategy and the feedback gain matrix method. In the latter, the control law of each vehicle is a linear combination of its states and states of the vehicle in front:

(2) $$u_{i}=K_{i,i}\zeta_{i}+K_{i,i-1}\zeta_{i-1},$$

where $\zeta_{i}=\left[\begin{array}{c} e_{pi}\left(t\right)\\ e_{vi}\left(t\right)\\ a_{i}\left(t\right) \end{array}\right]$ represents the vector of system states and $K_{i,j}$ represents a real array. The model presented in this section will be used in the design phase of the two control methods (i.e., DMPC strategy and feedback gain matrix method), but also to simulate the longitudinal dynamics of the platoon.

### 4.2. Communication Topologies

The use of vehicular communication in designing the CACC solution improves driving performance, safety, and stability. Vehicles can obtain information about velocities, accelerations, and positions from vehicles in front and use them to decide the new action so that the imposed constraints and targets are respected. The main advantages of V2V communication are represented by [19,119]: (i) improving safety, (ii) optimising the use of roads by reducing the space between vehicles, (iii) reducing fuel consumption and pollution by minimising the accelerations, (iv) improving control performances, and (v) ensuring string stability. The most studied communication typologies are represented by (i) predecessor–follower communication, where each follower *i* receives information from the vehicle in front i−1; (ii) leader–follower, where each follower *i* receives information from the leader vehicle i=0; (iii) leader–predecessor–follower, where each follower *i* receives information from the vehicle in front i−1 and from the leader i=0; (iv) bidirectional communication, where each vehicle *i* receives information from its neighbour vehicles i+1 and i−1. However, the performance of the vehicle platoons that use these communication topologies depends on the model and the chosen control solution. In the case of the MPC algorithm, some studies show that the control performances are quite similar for these topologies [68,120]. Also, solutions that ensure string stability and performances for a vehicle platoon’s lateral and longitudinal dynamics are proposed in [121,122,123]. These solutions use predecessor–follower communication in cases where the dynamics of a follower is described taking into account the position of the vehicle in front, and leader–follower communication when the dynamics of a follower is described taking into account the position of the leader or both of them.

Due to the fact that the model (1) describes the position of a vehicle to the vehicle in front and a follower *i* is coupled with follower i−1 through the acceleration, this work uses the predecessor–follower communication topology.

### 4.3. Distributed Model-Based Predictive Control Method

This section presents the DMPC strategy used to control the velocity and distance between vehicles. The algorithm is used by each vehicle to compute its control inputs (i.e., acceleration requests). The method uses a model of the vehicle to predict its behaviour and to determine a sequence of optimal control inputs so that a cost function is minimised. Also, due to the coupling between two consecutive vehicles, the method supposes communication between vehicles regarding their prediction of acceleration. With this, the prediction of vehicle future states is improved, which implies better performance.

Consider a system in chain architecture described by:

(3) $$\zeta_{i}\left(k+1\right)=A_{i}\zeta_{i}\left(k\right)+B_{i}u_{i}\left(k\right)+A_{i,i-1}\zeta_{i-1}\left(k\right),$$

where $A_{i}\in R^{n\times n}$, $B_{i}\in R^{m\times n}$, $A_{i,i-1}\in R^{n\times n_{i,i-1}}$, *n* represents the number of states for subsystem *i*, *m* represents the number of inputs for subsystem *i*, $n_{i,i-1}$ represents the number of states through which the subsystems *i* and i−1 are coupled, and i=0,…, M, M+1 represents the number of subsystems.

The optimal sequence of control inputs is determined by solving at each sample time the DMPC problem 1. This problem assumes the minimisation of a cost function that has three types of terms: (i) terms that minimise the prediction of vehicle position and velocity errors, (ii) terms that minimise the control efforts, and (iii) terms that minimise the error between the prediction of the states of vehicle $\zeta_{i}\left(\cdot \right)$ and the assumed prediction of the states of the vehicle in front ${\tilde{\zeta }}_{i-1}\left(\cdot \right)$. Moreover, the DMPC problem 1 takes into account the imposed constraints on vehicle inputs and states.

**Problem 1.**  *At each discrete step k, starting from an initial state $\zeta_{i}\left(k\right)=\zeta_{0}$ and using the system model* (3) *to predict the states of the vehicles, compute a finite horizon optimal input sequence minimising the cost function:*

(4) $$\begin{array}{ccc} J_{i}\left(k,\zeta_{i}\left(k\right),U_{i}\left(k\right)\right)= & \zeta_{i}{\left(N|k\right)}^{T}Q_{i}\zeta_{i}\left(N|k\right)+{\left(\zeta_{i}\left(N|k\right)-{\tilde{\zeta }}_{i-1}\left(N|k\right)\right)}^{T}W_{i}\left(\zeta_{i}\left(N|k\right)-{\tilde{\zeta }}_{i-1}\left(N|k\right)\right)+ & \\ & +\sum_{j=0}^{N-1}[\zeta_{i}{\left(j|k\right)}^{T}Q_{i}\zeta_{i}\left(j|k\right)+u_{i}^{T}\left(j|k\right)R_{i}u_{i}\left(j|k\right)+ & \\ & +{\left(\zeta_{i}\left(j|k\right)-{\tilde{\zeta }}_{i-1}\left(j|k\right)\right)}^{T}W_{i}\left(\zeta_{i}\left(j|k\right)-{\tilde{\zeta }}_{i-1}\left(j|k\right)\right)] \end{array}$$

*over $U_{i}\left(k\right)$, subject to the following constraints:*

(5) $$\begin{array}{c} U_{i}^{min}\leq U_{i}\left(j|k\right)\leq U_{i}^{max},\\ \zeta_{i}^{min}\leq \zeta_{i}\left(j|k\right)\leq \zeta_{i}^{max}, \end{array}$$

*where $Q_{i}$, $R_{i}$, and $W_{i}$ represent the weighting matrices, $W_{0}=0$ (for the leader), N represents the prediction horizon, $U_{i}\left(k\right)={\left[u_{i}\left(0|k\right),\ldots ,u_{i}\left(N-1|k\right)\right]}^{T}$ is the sequence of control inputs, and ${\tilde{\zeta }}_{i-1}={\left[\zeta_{i-1}\left(2|k-1\right),\ldots ,\zeta_{i-1}\left(N|k-1\right),\zeta_{i-1}\left(N|k-1\right)\right]}^{T}$ represents the prediction of the states for vehicle i−1, computed at step k−1 and sent to vehicle i.*

**Remark 1.**  *Note that for the leader vehicle, with index i=0, the vehicle in front is considered to be the “virtual leader” that is driving with the imposed acceleration, so ${\tilde{\zeta }}_{-1}$ represents the imposed acceleration for the leader for the next N steps.*

### 4.4. Feedback Gain Matrix Method

The second method uses a feedback gain matrix to compute the control inputs instead of a complex algorithm, such as DMPC. This solution has the advantage of requiring a low computational effort and being easier to be implemented on hardware with limited storage and computational capabilities. In what follows, the method used to determine this control matrix is detailed.

The model (3) can be rewritten as

(6) $$\zeta_{ℵ}\left(k+1\right)=A_{ℵ}\zeta_{ℵ}\left(k\right)+B_{ℵ}u_{ℵ}\left(k\right)+A_{ℵ,0}\zeta_{r}\left(k\right),$$

where $\zeta_{ℵ}={\left[\zeta_{0}^{T},\ldots ,\zeta_{M}^{T}\right]}^{T}$ and $u_{ℵ}={\left[u_{0}^{T},\ldots ,u_{m}^{T}\right]}^{T}$ aggregate all states and inputs of each subsystem i=0,…,M, $\zeta_{r}\in R^{n_{r}}$ represents the imposed reference for subsystem 0 (i.e., the leader), $A_{ℵ,0}=\left[\begin{array}{c} A_{n,n_{r}}\\ O_{n,n_{r}}\\ \ldots \\ O_{n,n_{r}} \end{array}\right]$, $O_{n,n_{r}}$ represents the null matrix of size $\left(n\times n_{r}\right)$.

Notice that the matrix $A_{n,n_{r}}$ corresponds to the matrix $A_{0,-1}$, and $\zeta_{r}$ to $x_{-1}$ from (3).

Then, the control law of the whole system can be defined as:

(7) $$u_{ℵ}\left(k\right)=K\zeta_{ℵ}\left(k\right),$$

where $K\in R^{\left(M+1)\times n(M+1\right)}$ represents the feedback gain matrix.

Moreover, each vehicle receives via V2V communication the states of the vehicle in front. Based on this, the control law of each follower is considered as in (2). The leader vehicle does not have a vehicle in front (except the virtual leader), so its control law is defined as $u_{0}=K_{0,0}\zeta_{0}$. The control matrix *K* has non-zero elements only on the sub-block (1,1) corresponding to the leader and on the sub-block {(i+1,i),(i+1,i+1)} corresponding to the follower vehicles. Notice that a sub-block (i,j) refers to the elements from matrix *K* represented by line *i* and columns {j,j+1,…,j+n−1}. Also, the notation $K_{i,j}$ is referring to the sub-block (i+1,j+1) in matrix *K*.

The control matrix *K* is obtained by solving Problem 2. The method assumes the use of a set of reference states, and the control matrix is computed so that the error between the reference states and model states (6) is minimised:

**Problem 2.**  *Starting from a set of reference states, compute the K matrix so that the following cost function is minimised:*

(8) $$\begin{array}{c} V\left(K\right)=\sum_{j=1}^{L}V_{j}\left(k\right), \end{array}$$

(9) $$V_{j}\left(k\right)=\sum_{k=0}^{t_{kf}}\left|\right|\zeta_{ℵ}^{r}\left(j,k\right)-\zeta_{ℵ}\left(k\right){\left|\right|}_{2}^{2},$$

*over K, subject to the following constraints:*

(10) $$\begin{array}{cc} \zeta_{ℵ}\left(k+1\right) & =A_{ℵ}\zeta_{ℵ}\left(k\right)+B_{ℵ}u_{ℵ}\left(k\right)+A_{ℵ,0}\zeta_{r}\left(j,k\right)\\ u_{ℵ}\left(k\right) & =K\zeta_{ℵ}\left(k\right),\\ \zeta_{ℵ}\left(0\right) & =\zeta_{ℵ}^{r}\left(j,0\right), \end{array}$$

*where $\zeta_{ℵ}^{r}$ represents the set of the reference states, $t_{kf}$ represents the length of a reference, L represents the number of states from the set.*

Note that the first solution, i.e., CACC based on the DMPC strategy, has the advantage that at each sample time, the method computes the command minimising a cost function that takes into account the prediction of the vehicle states, i.e., position and velocity errors and acceleration, as well as imposed constraints on the states and inputs, and also takes into account the information about the prediction of acceleration received from the vehicle in front. However, it has the disadvantage of requiring a high computing power to solve optimisation Problem 1. The second method requires a significant computational effort, but only in the phase of computing the feedback gain matrix *K*. After that, the command is computed at each sample time using Equation (2). The disadvantages of this method are represented by the possibility of violating imposed constraints, and also by the fact that it does not use a prediction of the vehicle’s state. Moreover, the method does not receive the prediction of the acceleration of the vehicle in front as it is using the method based on the DMPC approach. But the last disadvantage can be minimised in the phase of computing the matrix *K* by "training" the feedback gain matrix, so that the error between the two methods is minimised by choosing the set of references as the solution of the DMPC algorithm [124] (see Problem 2).

## 5. Illustrative Results

This section presents the simulation results obtained using the proposed control solutions. The platoon is formed by a leader, followed by three follower vehicles. As it was previously mentioned, the leader and follower vehicles use their sensors to obtain information about their velocity, acceleration, position, and velocity errors. These measurements are used by the control methods to obtain the prediction of vehicle states and to compute the control input. Also, the vehicles receive from their vehicle in-front information that contains the prediction of their accelerations (DMPC method) and their states (feedback gain matrix method). Note that the leader vehicle does not have a real vehicle in front, which means that model (1) can be used to compute the position and velocity error states.

**Figure 5:**
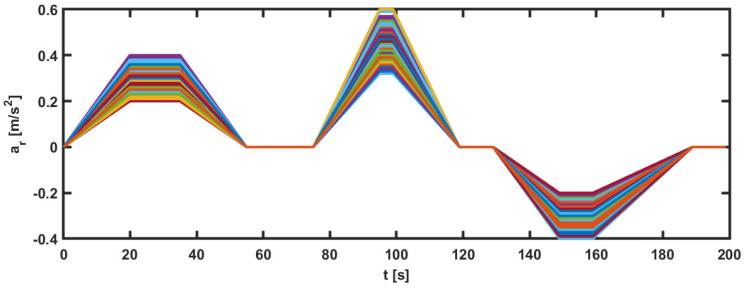
Set of 100 accelerations used by the second control method to design the control feedback matrix for the platoon.

The parameters used by the DMPC controller are represented by prediction horizon N=50 time samples, $Q_{0}=Q_{1}=\ldots =Q_{M}=diag\left\{1,10,0.1\right\}$, $R_{0}=R_{1}=\ldots =R_{M}=0.1$, $W_{0}=0$, $W_{i}=diag\left\{3,3,3\right\}$, i=1,…,M, M=3. The limits imposed on the input *u* and longitudinal error $e_{p}$ are represented by $u^{min}=-2$ m/s${}^{2}$, $u^{max}=2$ m/s${}^{2}$, $e_{pi}^{min}=-0.7$ m, $e_{pi}^{max}=0.7$ m. The used sample time is $T_{s}=0.1$ s. For the second method based on the feedback matrix, the set of the reference state is formed by L=100 references computed using the DMPC strategy. These reference states were obtained using a set of 100 reference accelerations illustrated in Figure 5. The length of each reference is $t_{kf}=200/T_{s}$.

**Figure 6:**
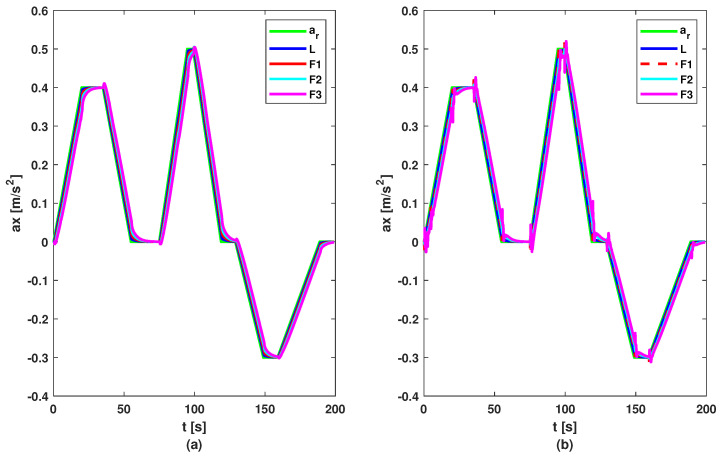
Accelerations of vehicles: (**a**) DMPC method; (**b**) feedback gain matrix method.

**Figure 7:**
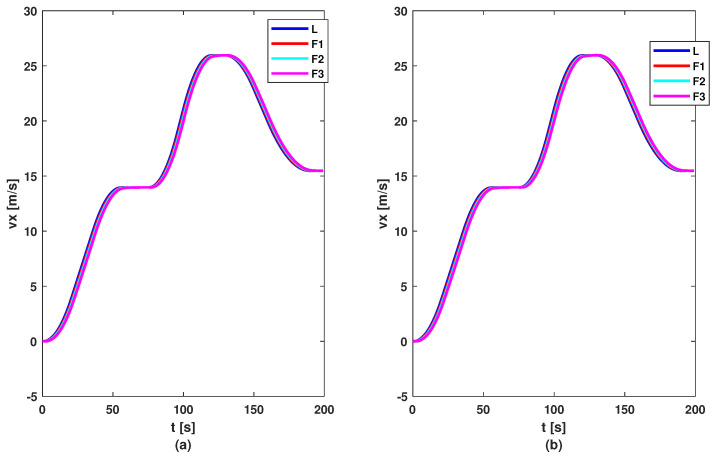
Velocities of vehicles: (**a**) DMPC method; (**b**) feedback gain matrix method.

**Figure 8:**
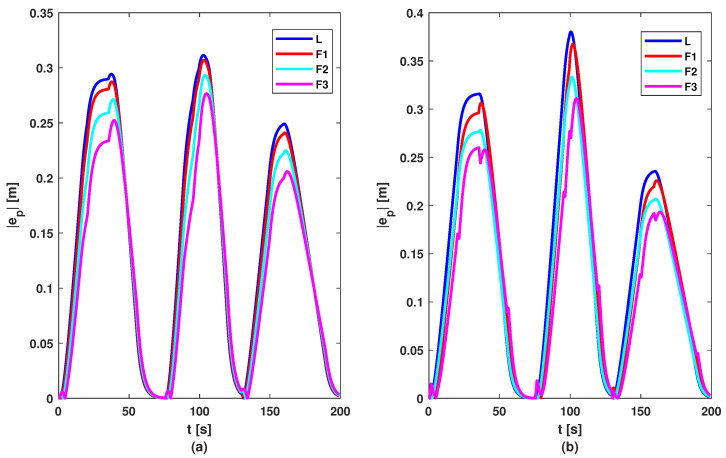
Position errors of vehicles: (**a**) DMPC method; (**b**) feedback gain matrix method.

**Figure 9:**
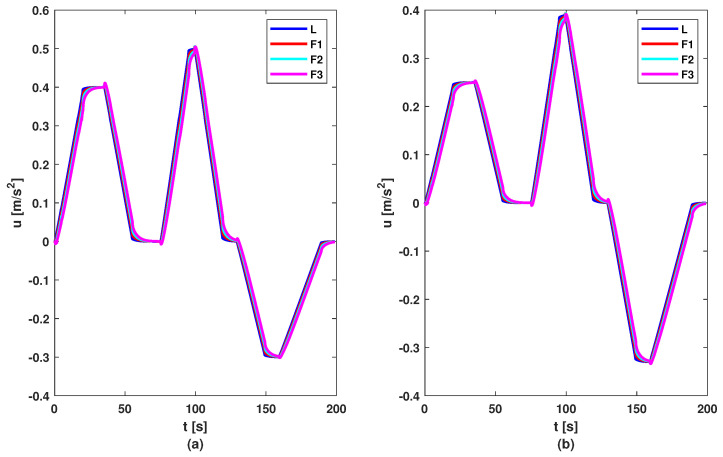
Control inputs of vehicles: (**a**) DMPC method; (**b**) feedback gain matrix method.

To test the proposed control methods, a simulation scenario was designed. The reference of the leader consists of a series of changes in imposed acceleration to test the methods in various situations. The reference and acceleration of vehicles are illustrated by Figure 6. Based on these figures, it can be noticed that all vehicles follow the acceleration and deceleration behaviour of the vehicle in front, but the first method (based on the DMPC algorithm) has a smoother acceleration compared to the second method (based on the feedback gain matrix). But this difference between methods is not noticed in the graph of velocity; see Figure 7, where all follower vehicles travel with the same velocity in both cases. All vehicles succeed in following the vehicle in front with imposed distance and with small errors, as can be observed in Figure 8. For all methods, the errors decrease in the upstream direction. Moreover, for the second method, the maximum of the absolute values of eigenvalues is $\rho =max(|A_{ℵ}+B_{ℵ}K|)=0.9774<1$, which means the platoon is globally stable. The control inputs are illustrated in Figure 9. The difference between the two methods is the following: the first method obtained a higher value for the requested acceleration compared to the second method, which implies higher fuel consumption. Also, all commands and position errors respect the imposed constraints.

**Remark 2.**  *Note that for each reference acceleration from those 100, the evolution of the states of the vehicle platoon was computed by imposing the reference accelerations for the platoon and having the DMPC strategy as a control solution. As a result, a set of 100 reference states, $\zeta_{ℵ}^{r}$, was obtained. The control feedback matrix K was computed by solving Problem 2. By finding a control law (7) so that the cost function (8) is minimised, the states of the platoon are led to follow the dynamics of the states controlled by the DMPC strategy. This means that the behaviour of the platoon (controlled with (7)) is close to the behaviour of the platoon controlled by the DMPC algorithm. Moreover, using a random large set of references, the platoon is tested in multiple operating points, ensuring that no bias from a particular case influences the calculus of matrix K.*

## 6. Discussion

**Figure 10:**
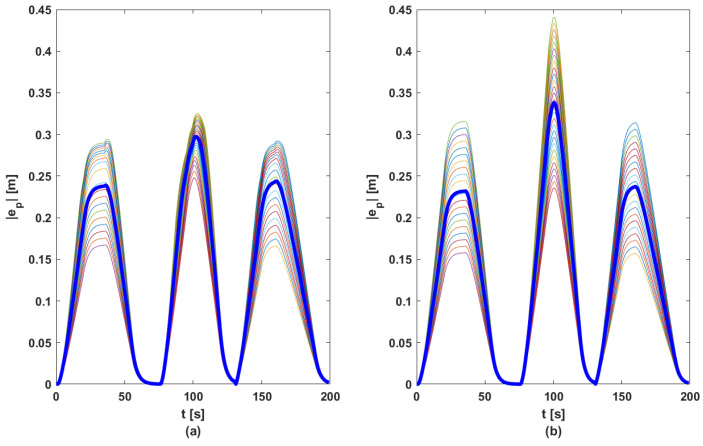
Position errors sets for L: (**a**) DMPC method; (**b**) feedback gain matrix method. Thin line—100 case; bold line—mean of $|e_{p}|$.

**Figure 11:**
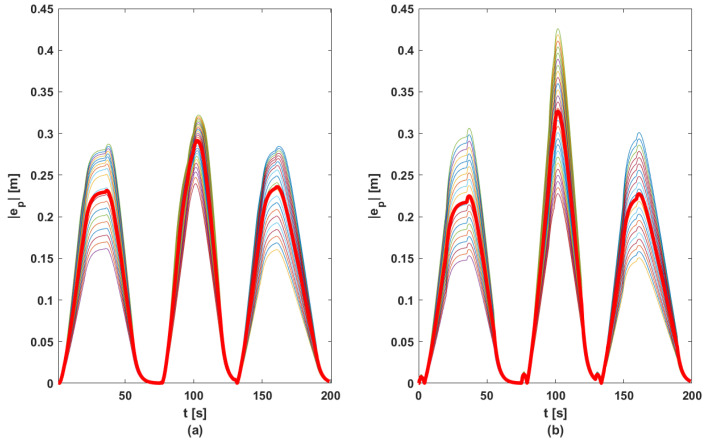
Position errors sets for F1: (**a**) DMPC method; (**b**) feedback gain matrix method. Thin line—100 case; bold line—mean of $|e_{p}|$.

**Figure 12:**
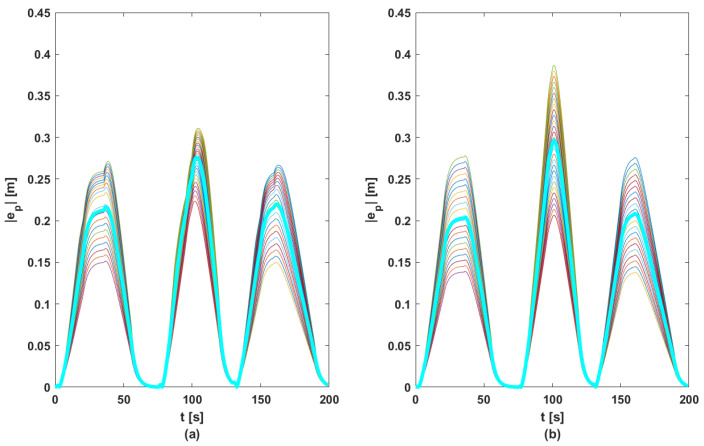
Position errors sets for F2: (**a**) DMPC method; (**b**) feedback gain matrix method. Thin line—100 case; bold line—mean of $|e_{p}|$.

**Figure 13:**
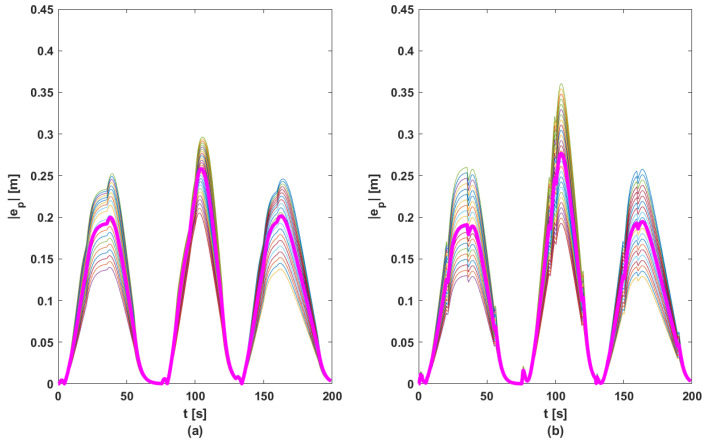
Position errors sets for F3: (**a**) DMPC method; (**b**) feedback gain matrix method. Thin line—100 case; bold line—mean of $|e_{p}|$.

In order to ensure a fair comparison between the two methods, each proposed control solution was tested for 100 random references for acceleration (other than those used in the phase of computing matrix *K*, see Figure 5). Figure 10, Figure 11, Figure 12 and Figure 13 contain the position error of the leader and follower vehicles obtained from 100 simulated cases. Moreover, for each vehicle, the average error is computed and illustrated with a black continuous line. From these result, it can be noticed that the maximum average position error for each vehicle are the following: (i) method based on DMPC algorithm—$max(e_{p0})=0.297$ m, $max(e_{p1})=0.291$ m, $max(e_{p2})=0.276$ m, $max(e_{p3})=0.258$ m; (ii) method based on feedback gain matrix—$max(e_{p0})=0.338$ m, $max(e_{p1})=0.326$ m, $max(e_{p2})=0.296$ m, $max(e_{p3})=0.276$ m.

Furthermore, using the results obtained from these 100 cases, a cumulative cost was used to evaluate better the performances of the two methods. This cost takes into account the position errors and input efforts of the vehicles:

(11) $$J=\frac{1}{t_{kf}}\sum_{s=1}^{100}\sum_{i=0}^{3}\sum_{j=1}^{t_{kf}}\left(e_{pi}^{2}\left(j\right)+u_{i}^{2}\left(j\right)\right).$$

The method based on DMPC obtains a cost equal to $J_{DMPC}=6.18$, and the second method, based on the feedback gain matrix, obtains a cost equal to $J_{K}=5.828$. Based on these results, the second method obtains control performances quite similar to the one based on the DMPC approach. As can be seen from these results, by designing the feedback matrix *K* using the proposed solution, it can be obtained a control solution that has a behaviour close to the DMPC algorithm but did not require specialised software and online optimisation to be implemented. These results made the second method proper for real-time implementation. The first method, based on the DMPC strategy, has the advantage that it can take into account online constraints imposed for the states and commands compared with the second method. Also, the first method, using a model of the system, can predict the state’s evolution. Although the used model usually has modelling errors, DMPC can obtain better performances. But the second control method is more suitable for real-time implementation because it does not require high computational power or complex optimisation algorithms to be implemented online.

The simulations were performed using MATLAB R2022b on Windows 10, 64-bit Operating System with a laptop Intel Core i7-8750H CPU @ 2.200 GHz and 8 GB RAM.

## 7. Conclusions and Future Work

This paper presents in detail the related levels of a highly advanced vehicle control architecture (sensing interface, perception, planning, decision, and control) and their element components. Moreover, the proposed control architecture was used to design a control solution for the longitudinal dynamics of a vehicle platoon. Also, for the control level, two methods were proposed: (i) the first one uses a complex control algorithm represented by DMPC; and (ii) the second method uses feedback gain matrices computed using results from the first method. The simulation results prove that the second method obtains similar performance compared to the one based on the DMPC algorithm, and this fact made it suitable for real-time implementation due to the simplicity of the control law.

Future work will focus on using the proposed control architecture in a laboratory scale, real-time vehicle platooning system. 

## Figures and Tables

**Table 1:** Comparison of different sensing technologies.

Metrics	Camera	Radar	LiDAR	Ultrasonic
Technology	Lights	Radio waves	Laser beams	Ultrasound waves
Range	≅250 m	5–200 m	≅200 m	Up to 10 m
Data per second	20–40 MB	10–100 KB	10–70 MB	10–100 KB
Bad weather functionality	Poor	Good	Fair	Good
Low lighting functionality	Fair	Good	Good	Good
Speed detection	Poor	Very good	Good	Poor
Distance detection	Poor	Very good	Good	Good
Resolution	Very good	Average	Good	Poor

**Table 2:** Classification of intra-vehicle network protocols.

Intra-Vehicle Network	Bit Rate	Data Length	Access Control	Messaging	Network Topology	Error Detection
LIN	19.2 Kb/s	8 bytes	Polling	Master–Slave	Bus	8-bit Checksum
CAN	125 Kb/s–1 Mb/s	0–8 bytes	CSMA/CA	Multi-Master	Bus Star	15-bit CRC
FlexRay	Up to 10 Mb/s	0–254 bytes	TDMA FTDMA	Multi-Master	Bus Star Multi-star	24-bit CRC
MOST	Up to 150 Mb/s	Up to 364 Bytes	TDMA Support for (a)synchronous	Master–Slave Streams	Ring	16-bit CRC
Automotive Ethernet	Up to 10 Gb/s	Up to 1500 bytes	CSMA/CR	Based on IP	Bus Star	32-bit CRC

**Table 3:** Comparison between communication standards used for V2X communication.

Features	DSRC	LTE-V2X	NR-V2X
Communication technology	IEEE 802.11p	LTE	5G NR
Frequency bands	5.9 GHz	5.9 GHz	5.9–52.6 GHz including mmWave
Data rates	3–27 Mb/s	150 Mb/s	1–10 Gb/s
Latency	Up to 150 ms	10–100 ms	<5 ms
Communication modes	Broadcast	Broadcast	Broadcast, unicast, and multicast
Mobility support	252 km/h	350 km/h	500 km/h
Transmission time	0.4 ms	1 ms	1 ms
Retransmission	None	Blind	HARQ-based
Sub-carrier spacing	156.25 KHz	15 KHz	Sub–6 GHz: 15, 30, 60 KHz; mmWave: 60, 120 KHz

## Data Availability

Not applicable.
